# Perceived stress of mothers and fathers on two NICUs before and during the SARS-CoV-2 pandemic

**DOI:** 10.1038/s41598-023-40836-9

**Published:** 2023-09-04

**Authors:** Philipp Deindl, Andrea Witting, Mona Dür, Angelika Berger, Katrin Klebermass-Schrehof, Dominique Singer, Vito Giordano, Renate Fuiko

**Affiliations:** 1 Department of Neonatology and Pediatric Intensive Care Medicine, University Children’s Hospital, University Medical Center Hamburg-Eppendorf, Hamburg, Germany; 2 Department of Pediatrics and Adolescent Medicine, Division of Neonatology, Pediatric Intensive Care and Neuropediatrics, Comprehensive Center for Pediatrics, Medical University of Vienna, Vienna, Austria; 3 Duervation, Krems, Austria; 4 Department of Neurobiology, Care Sciences and Society, Karolinska Institutet, Stockholm, Sweden

**Keywords:** Psychology, Medical research

## Abstract

Parents of very low birth weight (VLBW) infants in a neonatal intensive care unit experienced additional stress during the SARS-CoV-2 pandemic due to the related restrictions in hospital visiting policies. Our study aimed to compare parents' burdens before and during the pandemic. This survey included 121 parents of 76 VLBW infants in two European Level IV perinatal centers before and during the pandemic. We performed standardized parent questionnaires with mothers and fathers separately to evaluate their emotional stress and well-being. The pandemic worsened the emotional well-being of parents of VLBW infants, particularly of mothers. During the pandemic, mothers reported significantly higher state anxiety levels (48.9 vs. 42.9, *p* = 0.026) and hampered bonding with the child (6.3 vs. 5.2, 0 = 0.003) than before. In addition, mothers felt more personally restricted than fathers (6.1 vs. 5.2, *p* = 0.003). Fathers experienced lower levels of stress than mothers; they were equally burdened before and during the pandemic. Restrictions in visiting policies for families of VLBW infants during the SARS-CoV-2 pandemic have a significant negative impact on parental stress and should therefore be applied cautiously.

## Introduction

Parents of very low birth weight (VLBW) infants face numerous burdens^1–3^. Feelings of guilt and being overwhelmed by the unexpected end of the pregnancy, the sudden concern for a prematurely born vulnerable child who may suffer health and developmental problems, and own health issues can cause considerable stress. It can be challenging for parents to develop a close bond with their premature infant. Different studies showed that parental stress might have negative long-term consequences on the parent–child interaction and the child's development^4, 5^. Several studies examined the relationship between visitation policies^6^, quality of visits^7^, the opportunity for rooming-in^8^, and frequency of parental visits^9^ and overall well-being, parental child bonding and breastfeeding. When interviewed, parents unanimously conclude that flexible visitation times and proximity to the child influence their well-being^7^. A Finnish study showed an association between infrequent visits by parents and later behavioral problems in their prematurely born children^9^. Regular parental visits, proximity, and involvement in the care of the premature infant in the NICU may promote parental bonding and infant development. Parent-centered care concepts with the possibility of parental presence without restrictions, skin-to-skin care, and involvement of parents in their child's care are employed to address these difficulties in modern neonatal intensive care units (NICUs).

The SARS-CoV-2 pandemic required significant social restrictions, including more stringent hospital visiting policies at the beginning of the pandemic. In many NICUs, only one parent was allowed to visit the newborn. Moreover, parents reported significant insecurity and anxiety concerning even more restrictive measures^10–12^.

We had the unique opportunity to survey parents of VLBW infants before and during the pandemic and compare their burdens regarding anxiety, parenting stress, and social support.

## Methods

### Study design

We performed this survey in the NICUs of the Level-IV perinatal center of the University Children's Hospital, Medical University Vienna, Austria, and the Level-IV University Children’s Hospital, University Medical Center Hamburg-Eppendorf, Germany. Between February and May 2021, parents of VLBW infants (gestational age under 32 weeks) took part in this survey after approval of the local ethical review committees (Ethics Committee of the Medical University of Vienna, Austria, Nr.:1504/2020; and Ethics Committee of the Medical Association Hamburg, Germany, Nr.: PV7403). We reported the survey results according to the AAPOR Reporting guidelines.

The historical control group consisted of families of preterm infants who took part in a previous study about the occupational balance in parents of premature infants performed during 2018 and 2019 at the perinatal center of the Medical University Vienna using the same questionnaires (Ethics 1891/2015)^13^.

### Visiting restrictions

The visiting restrictions implemented during the SARS-CoV-2 pandemic stipulated that only one parent had access to their hospitalized child at a time in both centers, and other visitors were not allowed. Neither at the Medical University in Vienna nor at the University Medical Center Hamburg-Eppendorf were any time restrictions regarding parents' visits before the pandemic.

### Survey instruments

The study included three standardized parent questionnaires and an anamnesis for parents' characteristics. Mothers and fathers were invited to fill in the questionnaires separately. Parents who were unable to read German were excluded because only questionnaires in German were available. Questionnaires were administered to parents toward the end of their stay, and they were asked to complete them regarding their most stressful time. We explicitly instructed parents to reflect on the most stressful period during their infant's hospitalization, rather than referring to any other events in their lives.

The State-Trait Anxiety Inventory Form (STAI)^14^ consists of 40 self-report items measuring the presence and severity of current symptoms of parents’ anxiety and a general tendency toward anxiety. The STAI clearly distinguishes between the “State Anxiety” Scale, defined as the current state of anxiety, asking how parents are feeling “right now” (e.g., “I am calm” or “I feel rested”) on a 4-point Likert scale ranging from “not at all” to “very much so”. The “Trait Anxiety” Scale assesses the frequency of feelings of anxiety “in general” from “almost never” to “almost always” (e.g., "I feel like crying" or "I am content"). Higher scores indicate greater anxiety; a score of 20 corresponds to the absence and a score of 80 to the maximum intensity of a feeling of anxiety.

The Social Support Questionnaire (F-SozU by Sommer & Fydrich, 1989, 1991)^15^: The German self-report questionnaire is a 14-item short form and measures subjectively perceived or anticipated support from the social environment (e.g., “I have people who share my joys and sorrows with me.”). Parents answer the 14 items on a 5-point Likert scale ranging from "strongly disagree" to "strongly agree." The higher the scale value, the greater the perceived support from the social environment. Scores (percentile rank) above 25 are defined as unremarkable.

Parenting Stress Index (PSI): Parenting stress was assessed using the Parenting Stress Index, Third Edition^16^, is designed to evaluate the magnitude of stress in the parent–child system. Stress in the parenting domain results from impaired parental functioning, which reduces the resources available to parents in coping with parenting and demands. We used four of seven sources of parental stress, Parental Attachment (e.g., “It is sometimes difficult to figure out my child's needs”), Social Isolation (e.g., “I often feel on my own”), Health (e.g., “Over the last six months I have been very physically exhausted”), and Personal Limitation (e.g., "I sometimes feel restricted by the responsibilities of being a mother/father"). Questions were answered by parents on a Likert scale from "strongly agree" to "strongly disagree" in terms of current life stress. Stanine values were then calculated with a range between one and nine. A value of seven and greater indicates a high stress level in this section.

### Data analysis

Continuous variables were expressed as mean ± standard deviation (SD) and range. Discrete data were compared between groups with the Fishers' exact, and the Pearson’s Chi-square tests. A two-tailed t-test was used to compare continuous variables before and after the intervention. In addition, we calculated linear regression models to analyze the impact of the predictor variables survey time point, CRIB II^17^, parent gender, parent marital status to analyze the parent's Social Support, Parenting Stress Index (including the subcategories bonding, personal restrictions, social integration, and health), and the STAI State and Trait Anxiety. P values less than 0.05 were considered significant. Statistical analyses were performed using R 4.1.2 (R Core Team, Vienna, Austria).

### Statement of ethics

This is a study was approved by the local ethics committee (ethic number: 1504/2020) of the Medical University of Vienna.

### Patient consent statement

Informed consent was obtained by each participants participating in this study.

## Results

### Infant characteristics

Overall, parents of 76 VLBW infants were included in the analysis. Thirty VLBW infants with a mean ± SD (range) gestational age of 27.1 ± 2 (23.3–31.4) weeks and a birthweight of 934 ± 295 (500–1490)g were examined in Vienna before the pandemic, and a total of 46 VLBWs with a mean ± SD gestational age of 26.8 ± 2.5 (22.9–31.7) weeks and a birthweight of 893 ± 309 (390–1500)g during the pandemic (35 in Vienna and 11 in Hamburg). Infant characteristics between the centers were similar (supplementary Table 1), therefore further analyses will refer to the aggregation of both groups.

Infant characteristics before and during the pandemic are presented in Table 1 showing no significant differences within descriptive characteristics and clinical outcomes.

**Table 1:** Infants characteristics.

Variable	Before COVID-19 pandemic	During COVID-19 pandemic	*p* value
	N = 30	N = 46	
Gestational age (weeks)	27.1 ± 2.0 (23.3–31.4)	26.8 ± 2.5 (22.9–31.7)	0.611
Birth weight (g)	934 ± 295 (500–1490)	893 ± 309 (390–1500)	0.562
Female gender	11 (36.7)	14 (30.4)	0.623
5' APGAR	8.4 ± 1.1 (4–9)	8.1 ± 1.6 (0–9)	0.399
CRIB II	9.6 ± 3.3 (3–16)	10.6 ± 4.2 (2–19)	0.248
Intraventricular hemorrhage Grade III or IV	3 (10)	6 (13)	1.0
Periventricular leukomalacia	1 (3.3)	1 (2.2)	1.0
Chronic lung disease	4 (13.3)	7 (15.2)	0.526
Retinopathy of prematurity Grade III or IV	2 (6.7)	1 (2.2)	0.558
Necrotizing enterocolitis	5 (16.7)	4 (8.7)	0.306
Length of hospital stay (days)	81.3 ± 24.2 (48–163)	95.0 ± 43.3 (41–250)	0.082

Continuous variables are shown as mean ± standard deviation and (range) and compared using a two-sided Welch Two Sample t-test. Categorical variables are shown as n (%) and compared using the Fisher's exact test.

### Parent characteristics

**Figure 1:**
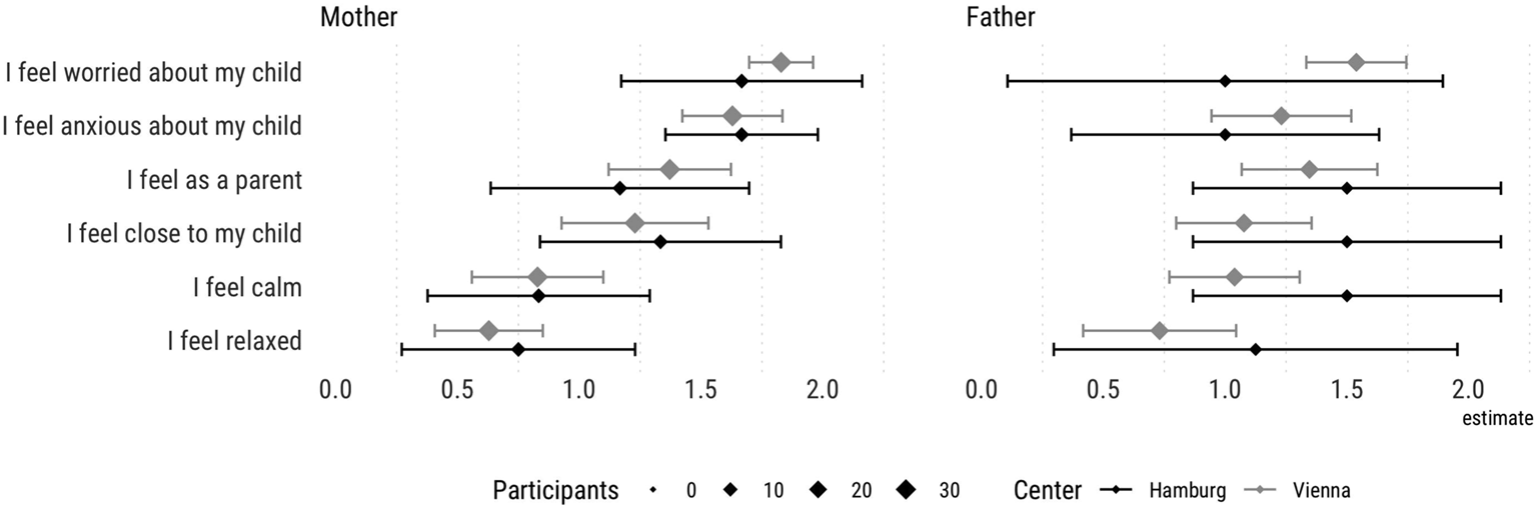
General description of parental feeling, in both centers, by the time questionnaires were completed.

We surveyed 121 parents of VLBW infants, 40 parents from Vienna before the pandemic and 81 parents during the pandemic (61 from Vienna, 20 from Hamburg). Table 2 shows detailed characteristics of the participating parents. Out of the total number of children, both parents responded in the case of 45 children. Among these 45 children, 10 pairs of parents completed the questionnaire before the onset of the pandemic, while 35 pairs of parents completed it during the pandemic. Figure 1 provides descriptive information about the actual feelings of mothers and fathers when the questionnaires were filled in. Parents’ characteristics were similar before and during the pandemic and between centers (see supplementary Table 2).

**Table 2:** Parents characteristics.

Variable	Before COVID-19 Pandemic	During COVID-19 Pandemic	*p* value
	N = 40	N = 81	
Gender			
Female	30 (75)	47 (58)	0.104
Male	10 (25)	34 (42)	
Age	32.7 ± 4.6 (22–42)	33.2 ± 4.8 (24–45)	0.612
Marital status			
Single	13 (32.5)	13 (16)	0.1
Married, registered partnership, living together	25 (62.5)	65 ( 80.2 )	
Divorced	2 (5)	3 (3.7)	
Highest level of education			
General school or Polytechnic school	1 (2.5)	6 (7.4)	0.76
Apprenticeship certificate	4 (10)	9 (11.1)	
Vocational school	5 (12.5)	5 (6.2)	
Grammar school (AHS, BHS)	10 (25)	23 (28.4)	
University or University of Applied Sciences	19 (47.5)	36 (44.4)	
Other/No Answer	1 (2.5)	2 (2.5)	

Continuous variables are shown as mean ± standard deviation and (range) and compared using a two-sided Welch Two Sample t-test. Categorical variables are shown as n (%) and compared using the Pearson's Chi-squared test with Yates' continuity correction.

### Parents’ burden

**Figure 2:**
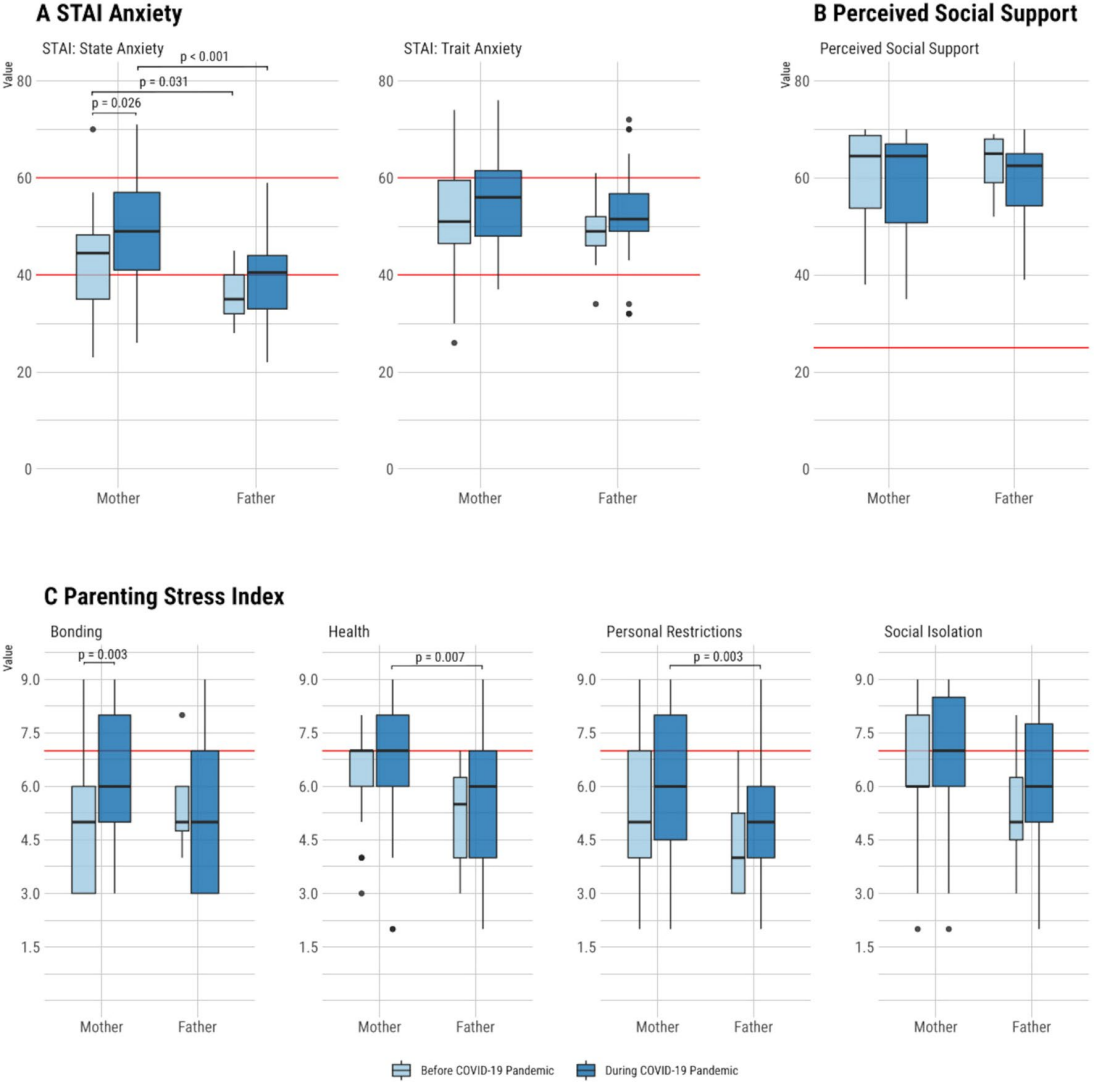
Survey results for (**A**) Anxiety, (**B**) Perceived Social Support, (**C**) Parenting Stress before and during the pandemic among parents of VLBWIs shown separately for mothers and fathers, respectively. The red lines indicate the lower and upper cutoff values for the respective scores.

Survey results of parents' burden are presented before and during the pandemic and shown separately for mothers and fathers (Fig. 2, supplementary Table 3).

### Anxiety (A)

**Figure 3:**
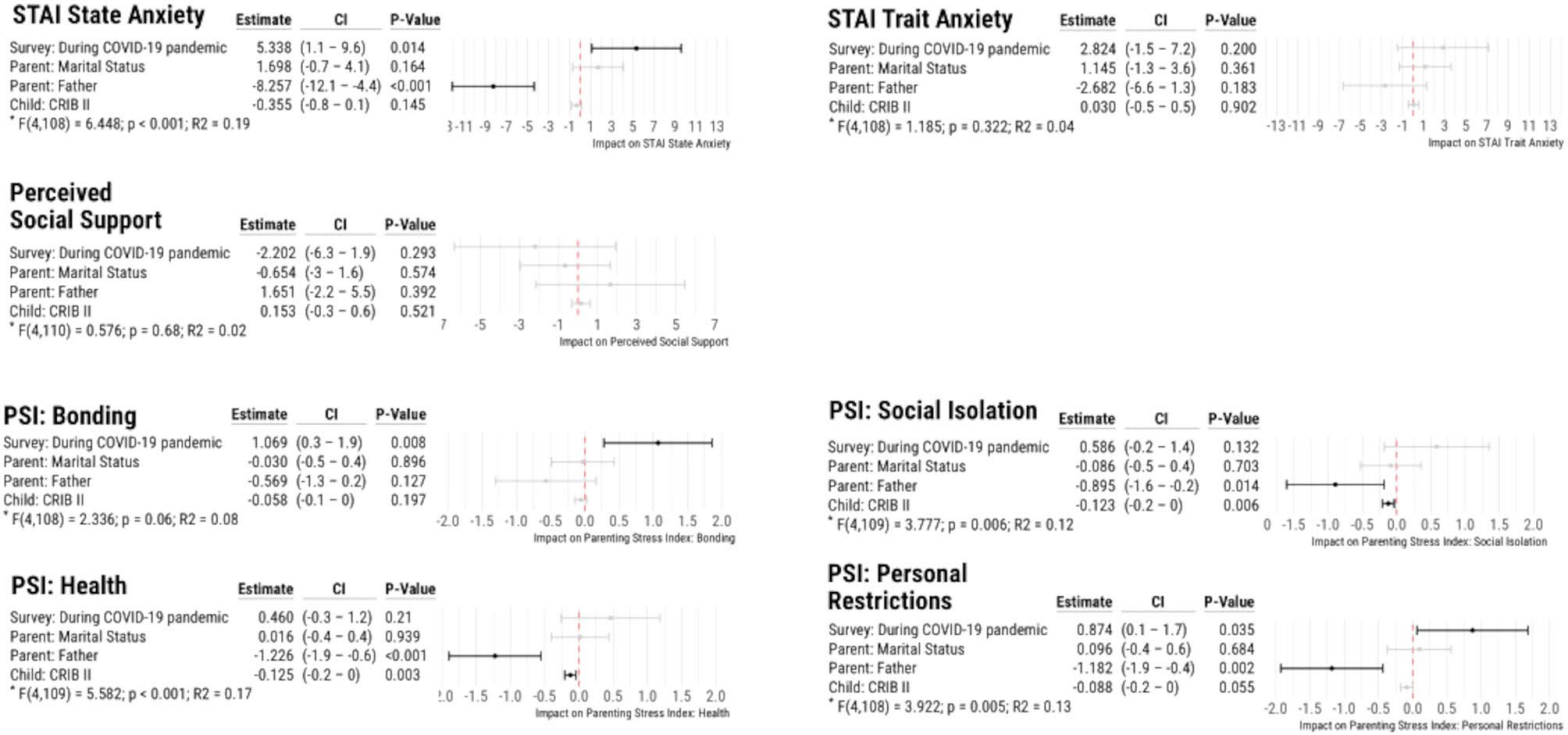
Numeric results, model performance indicators and plots for each of the linear regression models calculated to analyze the impact of the predictors survey time point, parent marital status, parent gender, and CRIB II on (**A**) State and Trait Anxiety, (**B**) Perceived Social Support, and (**C**) Parenting Stress subcategories. CI: 95% confidence interval, CRIB II: Critical Risk Index for Babies II, R2: R-squared.

According to the STAI State Questionnaire, state anxiety was significantly higher among mothers during the pandemic (48.9 ± 11.0) than before (42.9 ± 10.1, *p* = 0.026). It was additionally more pronounced among mothers than among fathers both before (42.9 ± 10.1 vs. 36.3 ± 6.0, *p* = 0.031) and during (48.93 ± 11.0 vs. 39.8 ± 9.6, *p* < 0.001) the pandemic (Fig. 2-A; supplementary Table 3). Linear regression models also showed that the pandemic significantly increased state anxiety, confirming that fathers reported lower anxiety scores on average than mothers (Fig. 3). On the other hand, trait anxiety did not differ between fathers and mothers according to the STAI trait questionnaire (Fig. 2-A). Furthermore, the linear regression model showed no impact of the respective predictors (survey time point, CRIB II, sex of the parent gender, parent marital status) analyzed on the trait anxiety of the parents (Fig. 3).

### Perceived social support (B)

Mothers and fathers reported similar perceived social support before and during the pandemic (Fig. 2-B, supplementary Table 3). This result was further confirmed by the linear regression model (Fig. 3).

### Parenting stress (C)

For mothers, bonding was more challenging during the pandemic than before (6.3 ± 1.8 vs. 4.9 ± 1.8, *p* = 0.003) (Fig. 2-C, supplementary Table 3). In addition, the linear regression model confirmed that the pandemic negatively influenced bonding, whereas the other tested predictors were not statistically significant (Fig. 3).

Mothers felt more personally restricted during the pandemic than fathers (6.1 ± 2.0 vs. 5.2 ± 2.0, *p* = 0.003) (Fig. 2-C, supplementary Table 3). The linear regression model confirmed that the pandemic significantly increased feelings of personal restriction, with fathers feeling less restricted than mothers (Fig. 3). In addition, mothers rated health burdens higher during the pandemic than fathers (6.6 ± 1.6 vs. 5.3 ± 1.5, *p* = 0.007).

In general, the linear regression model also revealed that greater severity of illness in children, indicated by a higher CRIB II score, led to lower ratings of social integration and a lower health burden rating (Fig. 3).

## Discussion

This survey at two Level IV perinatal centers in German-speaking countries shows that the SARS-CoV-2 pandemic significantly impacted the burden on parents of high-risk preterm infants. Parents perceived more anxiety, and suffered from personal restrictions and impaired bonding with their children following visiting restrictions during the pandemic. In addition, we detected gender differences among parents, with fathers reporting less interference for each aspect.

Premature neonates require specialized care in the Neonatal Intensive Care Unit (NICU) due to their unique medical needs. While medical interventions play a crucial role in their survival and well-being, parental support and presence have emerged as essential components of their care. Parental involvement for premature neonates in the NICU has a significant impact on their long-term development due to several crucial aspects of parent infant interaction: (1) emotional Bonding: Parental presence allows premature neonates to develop a secure attachment, facilitating emotional bonding and fostering a sense of security and trust^18, 19^; (2) neurodevelopment: Parental support, including skin-to-skin contact (kangaroo care), promotes neurodevelopmental outcomes by reducing stress, improving physiological stability, and enhancing brain development^20^; (3) breastfeeding: Parental presence encourages breastfeeding, which provides essential nutrients, immune factors, and promotes infant growth and development; (4) Long-term Outcomes: Numerous studies have demonstrated that parental support and presence in the NICU lead to improved cognitive, social, and emotional outcomes in premature neonates during infancy and later in life^20, 21^.

Our ﻿results﻿ show that additional negative impact on parental stress resulted from visitation restrictions during the pandemic. In detail, we observed a significant increase in self-reported anxiety in mothers of preterm infants before and during the pandemic, consistent with the literature^12,22, 23^, reporting increased maternal stress during the pandemic. Emotionally distressed parents find it more challenging to fulfill their roles in caring for, supporting, and raising their infant^24^. This is of particular relevance as parental support is extremely important for children hospitalized in an intensive care unit^21,25, 26^. It has been shown that hospitalized infants thrive best when they are consistently cared for by their parents in a family-integrated setting^25^. Parental presence has been found to be associated with benefits for preterm infants, including more stable physiological responses, improved oral feeding, and reduced length of stay^25, 26^. The more stressed and burdened parents are, the less well they can be supportive for their children. Therefore, the additional stress caused by the pandemic may have a significant impact on parents but also on their premature infant.

We also found less bonding in mothers with their preterm infant during the pandemic, while the father-child attachment was mainly unaffected. Parent-infant bonding plays a crucial role in fostering a positive parent-infant relationship and promoting long-term infant health^27, 28^. However, the experience of preterm birth can disrupt or delay this bonding process^29^. Studies have shown that bonding is influenced by factors such as physical proximity between parent and infant, the emotional state of the mother, and the infant's ability to communicate^27^. Unfortunately, these aspects can be compromised in the neonatal intensive care setting. Our results show that visitation restrictions in the context of the SARS-CoV-2 pandemic were an additional disruptive factor for mother–child bonding. Problems regarding attachment in mother-preterm dyads during the pandemic have been reported before^22–24,30^. Knowing that bonding in the first few weeks of a child's life is of great importance, these infants might suffer from long-term consequences in their emotional but also neurological development.

When comparing mothers and fathers, findings indicate a significant difference as mothers experienced health stress, loss of energy, and psychological and physical exhaustion much more than fathers. Our results suggest that the physical environment of the NICU becomes an even more stressful setting during the pandemic, associated with health issues, personal restrictions, and lower levels of attachment, especially among mothers. This is in line with the study of Hagen et al.^31^ demonstrating that mothers experience more stress when being with the baby in the NICU without the father. We interpret this finding in our study as a consequence of the mother spending more time in the ward and the missing supporting role of the father or other relatives. During the pandemic, parents were not allowed to stay in the NICU together at the same time. These restrictions allowed closeness between the child and only one of the two parents and hampered emotional moments and mutual support as a couple under challenging situations. The only exception was the first week after birth, when mothers were still admitted to the postnatal ward. Thus, it can be derived from the results of our study, that the supportive role of fathers and other family members for both infants’ care and mothers’ support is important, even in times of a pandemic. It will be essential to further develop parent-focused interventions during a NICU stay also for times of pandemic situations, mainly to improve attachment quality and health outcomes and reduce personal limitations, stress, and anxiety^25,26,32^. Erdei and Liu^33^ published guidelines for supporting family-well-being in the NICU during a pandemic. Kostenzer et al.^34^ underlined the importance of parental presence for infants’ well-being in a NICU setting and published a call for “zero separation”.

There are some strengths of this study that merit special attention. This is one of few studies with a historical control group focusing on parents' experiences with infants in need of intensive care two years before the pandemic. In addition, we were able to comprehensively describe mothers' and fathers' experiences of stress in the NICU. In most previous studies, parents were interviewed together, or the number of fathers who participated in the study was low. In the current study, we were able to interview both, father and mother, separately, to highlight the differences between the parents. We also paid particular attention to the selection of questionnaires to ensure well-validated and internationally recognized questionnaires standardized on a large sample, thus avoiding self-designed and non-comparable questionnaires.

There are some limitations of this study that need to be considered. Conducting surveys in only two perinatal hospitals carries the risk of selection bias and may have affected the representativeness of participants. Only parents who were able to read and understand German could participate in the study. Probably we missed parents with most burden due to multiple risk factors (migration, social integration, financial problems). Nevertheless, we found that the characteristics of both high-risk infants and parents did not differ between Vienna and Hamburg, so we assume that study subjects are very likely to have been comparable between the two centers also before the pandemic.

## Conclusions

This study showed a substantial negative impact of the SARS-CoV-2 pandemic on multiple aspects of burden and stress among parents of VLBW infants. Potential negative long-term consequences for parents and infants following the restrictions during the pandemic are not yet known. Caregivers should be aware that families of VLBW infants are a vulnerable population experiencing multiple traumas and stress during their time in the NICU and that additional stress factors such as visiting restrictions increase this burden and stress. In view of the future, the elaboration of developmental care guidelines in times of pandemics is warranted, facilitating the presence of both parents in the NICU and communication between parents as important aspects for copying with a very stressful event such as a preterm birth. Further studies should focus on the potential adverse long-term consequences of this stressful experience on infants and families.

### Supplementary Information

Supplementary Table 1.

Supplementary Table 2.

Supplementary Table 3.

## Data Availability

All data generated or analysed during this study are included in this published article (and its supplementary information files). The datasets generated during and/or analysed during the current study are available from the corresponding author on reasonable request.
