# Supplementary material for: Perceived stress of mothers and fathers on two NICUs before and during the SARS-CoV-2 pandemic
Source: Sci Rep. 2023 Sep 4;13:14540. doi: 10.1038/s41598-023-40836-9 (PMC10477236; doi:10.1038/s41598-023-40836-9)
Supplement: Supplementary file 1 — Supplementary Table 1. [file 41598_2023_40836_MOESM1_ESM.docx]

| **Supplementary Table 1: Infants characteristics during COVID-19 pandemic according to center** | | | | |  |  |
| --- | --- | --- | --- | --- | --- | --- |
| **Variable** |  | **Vienna (Austria)** |  | **Hamburg (Germany)** |  | **P-Value** |
|  |  | **N = 35** |  | **N = 11** |  |  |
| Gestational age (weeks) |  | 26.7 ± 2.4 ( 22.9–31.7 ) |  | 27.2 ± 2.9 ( 23.9–31.4 ) |  | 0.601 |
| Birth weight (g) |  | 868.4 ± 298.5 ( 480–1500 ) |  | 969.1 ± 341.6 ( 390–1400 ) |  | 0.394 |
| Female gender |  | 11 (36.7) |  | 14 (30.4) |  | 0.713 |
| 5' APGAR |  | 8.2 ± 1.7 ( 0–9 ) |  | 7.9 ± 1 ( 6–9 ) |  | 0.542 |
| CRIB II |  | 10.8 ± 4.2 ( 2–19 ) |  | 9.9 ± 4.3 ( 4–16 ) |  | 0.567 |
| Intraventricular hemorrhage Grade III or IV |  | 6 (17) |  | 0 (0) |  | 0.311 |
| Periventricular leukomalacia |  | 0 (0) |  | 1 (9) |  | 0.239 |
| Chronic lung disease |  | 7 (20) |  | 3 (27.3) |  | 0.682 |
| Retinopathy of prematurity Grade III or IV |  | 1 (2.9) |  | 0 (0) |  | 1.0 |
| Necrotizing enterocolitis |  | 3 (8.6) |  | 1 (9.1) |  | 1.0 |
| Length of hospital stay (days) |  | 97.7 ± 44.8 ( 41–250 ) |  | 86.5 ± 39 ( 43–155 ) |  | 0.430 |
| Continuous variables are shown as mean ± standard deviation and (range) and compared using a two-sided Welch Two Sample t-test. Categorical variables are shown as n (%) and compared using the Fisher's exact test. | | | | | | |
