# Supplementary material for: Perceived stress of mothers and fathers on two NICUs before and during the SARS-CoV-2 pandemic
Source: Sci Rep. 2023 Sep 4;13:14540. doi: 10.1038/s41598-023-40836-9 (PMC10477236; doi:10.1038/s41598-023-40836-9)
Supplement: Supplementary file 2 — Supplementary Table 2. [file 41598_2023_40836_MOESM2_ESM.docx]

| **Supplementary Table 2: Parents characteristics during COVID-19 pandemic according to center** | | | | |  |  |
| --- | --- | --- | --- | --- | --- | --- |
| **Variable** |  | **Vienna (Austria)** |  | **Hamburg (Germany)** |  | **P-Value** |
|  |  | **N = 61** |  | **N = 20** |  |  |
| Gender |  |  |  |  |  |  |
| Female |  | 29 ( 58 ) |  | 12 (60) |  | 1.000 |
| Male |  | 21 ( 42 ) |  | 8 ( 40 ) |  |  |
| Age |  | 32.6 ± 4.7 (24–43) |  | 35.0 ± 4.9 (27–45) |  | 1.000 |
| Marital status |  |  |  |  |  |  |
| Single |  | 8 ( 16 ) |  | 5 ( 25 ) |  | 0.193 |
| Married, registered partnership, living together |  | 41 ( 82 ) |  | 13 ( 65 ) |  |  |
| Divorced |  | 1 ( 2 ) |  | 2 ( 10 ) |  |  |
| Highest level of education |  |  |  |  |  |  |
| General school or Polytechnic school |  | 1 ( 2 ) |  | 5 ( 25 ) |  | 0.063 |
| Apprenticeship certificate |  | 5 ( 10 ) |  | 2 ( 10 ) |  |  |
| Vocational school |  | 2 ( 4 ) |  | 1 ( 5 ) |  |  |
| Grammar school (AHS, BHS) |  | 15 ( 30 ) |  | 4 ( 20 ) |  |  |
| University or University of Applied Sciences |  | 25 ( 50 ) |  | 8 ( 40 ) |  |  |
| Other / No Answer |  | 2 ( 4 ) |  | – |  |  |
| Continuous variables are shown as mean ± standard deviation and (range) and compared using a two-sided Welch Two Sample t-test. Categorical variables are shown as n (%) and compared using the Pearson's Chi-squared test with Yates' continuity correction. | | | | | | |
