# Supplementary material for: Perceived stress of mothers and fathers on two NICUs before and during the SARS-CoV-2 pandemic
Source: Sci Rep. 2023 Sep 4;13:14540. doi: 10.1038/s41598-023-40836-9 (PMC10477236; doi:10.1038/s41598-023-40836-9)
Supplement: Supplementary file 3 — Supplementary Table 3. [file 41598_2023_40836_MOESM3_ESM.docx]

| **Supplementary Table 3: Survey results** | | | | | | | | |
| --- | --- | --- | --- | --- | --- | --- | --- | --- |
| **Variable** |  | **Parent** |  | **Before COVID–19 Pandemic** |  | **During COVID–19 Pandemic** |  | **P-Value** |
|  |  |  |  | **N = 40** |  | **N = 81** |  |  |
| STAI |  |  |  |  |  |  |  |  |
| State Anxiety |  | Mother |  | 42.9 ± 10.1 (23–70) |  | 48.9 ± 11 (26–71) |  | **0.026** |
| State Anxiety |  | Father |  | 36.3 ± 6 (28–45) |  | 39.8 ± 9.6 (22–59) |  | 0.193 |
| Trait Anxiety |  | Mother |  | 52.1 ± 11.6 (26–74) |  | 55.1 ± 9.7 (37–76) |  | 0.287 |
| Trait Anxiety |  | Father |  | 48.9 ± 8.3 (34–61) |  | 52.4 ± 10.3 (32–72) |  | 0.303 |
| Social Support |  |  |  |  |  |  |  |  |
| Perceived Social Support |  | Mother |  | 60.4 ± 10.6 (38–70) |  | 58.5 ± 11.3 (35–70) |  | 0.487 |
| Perceived Social Support |  | Father |  | 62.9 ± 6.1 (52–69) |  | 60 ± 7.4 (39–70) |  | 0.241 |
| Parenting Stress Index |  |  |  |  |  |  |  |  |
| Bonding |  | Mother |  | 4.9 ± 1.8 (3–9) |  | 6.3 ± 1.8 (3–9) |  | **0.003** |
| Bonding |  | Father |  | 5.4 ± 1.3 (4–8) |  | 5.4 ± 2 (3–9) |  | 0.99 |
| Health |  | Mother |  | 6.2 ± 1.3 (3–8) |  | 6.6 ± 1.6 (2–9) |  | 0.296 |
| Health |  | Father |  | 5.2 ± 1.5 (3–7) |  | 5.4 ± 2.3 (2–9) |  | 0.877 |
| Personal Restrictions |  | Mother |  | 5.2 ± 2 (2–9) |  | 6.1 ± 2 (2–9) |  | 0.082 |
| Personal Restrictions |  | Father |  | 4.4 ± 1.5 (3–7) |  | 4.8 ± 1.8 (2–9) |  | 0.478 |
| Social Isolation |  | Mother |  | 6.4 ± 1.8 (2–9) |  | 6.7 ± 1.9 (2–9) |  | 0.532 |
| Social Isolation |  | Father |  | 5.2 ± 1.8 (3–8) |  | 6 ± 1.9 (2–9) |  | 0.326 |
| Continuous variables are shown as mean ± standard deviation and (range). | | | | | | | | |
